# Model-Based Evaluation of HangAmDan-B1 and Afatinib Combination Therapy in HCC827 Xenograft Mice with Resistance to Epidermal Growth Factor Receptor Tyrosine Kinase Inhibitor

**DOI:** 10.3390/ph18050748

**Published:** 2025-05-19

**Authors:** Sung-yoon Yang, Lien Thi Ngo, Soyoung Lee, Hwi-yeol Yun, Tham Thi Bui, Dong-Hyeon Kim, Jung-woo Chae, Sojung Park

**Affiliations:** 1 College of Pharmacy, Chungnam National University, Daejeon 34134, Republic of Korea; 201851000@o.cnu.ac.kr (S.-y.Y.); sy.lee@cnu.ac.kr (S.L.); hyyun@cnu.ac.kr (H.-y.Y.); bttham@hpmu.edu.vn (T.T.B.); 2 Faculty of Pharmacy, PHENIKAA University, Yen Nghia, Ha Dong, Hanoi 12116, Vietnam; lien.ngothi@phenikaa-uni.edu.vn; 3 PHENIKAA Research and Technology Institute (PRATI), A&A Green Phoenix Group JSC, No. 167 Hoang Ngan, Trung Hoa, Cau Giay, Hanoi 11313, Vietnam; 4 Department of Bio-AI Convergence, Chungnam National University, Daejeon 34134, Republic of Korea; 5 Senior Health Convergence Research Center, Chungnam National University, Daejeon 34134, Republic of Korea; 6 Faculty of Pharmacy, Haiphong University of Medicine and Pharmacy, Haiphong 180000, Vietnam; 7 Department of Internal Medicine, Pusan National University, Korean Medicine Hospital, Yangsan 50612, Republic of Korea; dongxian92@gmail.com; 8 Department of Korean Internal Medicine, School of Korean Medicine, Pusan National University, Yangsan 50612, Republic of Korea

**Keywords:** pharmacokinetic/pharmacodynamic modeling, afatinib, hangamdan-B1, non-small cell lung cancer, EGFR-TKI resistance, drug–drug interactions

## Abstract

**Objectives:** HangAmDan-B1 (HAD-B1), a blended herbal mixture, has been investigated as an adjuvant therapy with afatinib (AFT) to treat non-small lung cancer (NSCLC). Although preclinical studies demonstrated promising synergistic results, clinical trials have not yet confirmed the expected benefits. This study aims to quantitatively examine the exposure–response relationship and synergistic interactions through pharmacokinetic/pharmacodynamic (PK/PD) modeling. **Methods:** A PK/PD model was established and validated based on tumor growth profiles from a xenograft mouse study of gefitinib-resistant HCC827. Model-based simulations were performed to predict and assess therapeutic effects across different treatment groups. **Results:** The PK/PD model confirmed HAD-B1 enhances the potency of AFT by 1.45-fold. Model-based simulations predicted that combination treatment maintains a lower tumor size compared to AFT monotherapy. **Conclusions:** This study quantitatively demonstrated the synergistic interaction between HAD-B1 and AFT. The developed PK/PD model provides insights into potential dosing strategies to treat NSCLC resistant to EGFR-TKIs. Further clinical trials are warranted to validate these findings and refine dosing strategies to improve therapeutic outcomes.

## 1. Introduction

Lung cancer continues to be the leading cause of cancer-related mortality globally [1]. Non-small cell lung cancer (NSCLC) accounts for approximately 85% of all lung cancer cases [2]. Epidermal growth factor receptor (EGFR) mutations are commonly found in patients with NSCLC, making EGFR tyrosine kinase inhibitors (TKIs) a cornerstone of targeted therapy [2,3]. Although patients initially respond well to EGFR-TKIs, they inevitably develop acquired resistance (AR), necessitating alternative treatments or investigative strategies. MET amplification is a well-recognized mechanism of AR to EGFR-TKIs, occurring in approximately 10–15% of patients with NSCLC treated with first- and second-generation EGFR-TKIs [4,5,6,7]. Combination therapies with MET inhibitors and EGFR-TKIs have been investigated to improve therapeutic efficacy and overcome drug resistance [8,9].

HangAmDan-B1 (HAD-B1) is a blended herbal extract developed as an adjunct therapy for NSCLC, aiming to augment the efficacy of EGFR-TKIs. The therapeutic potential of HAD-B1 across various settings has been demonstrated in several preclinical studies. Kang et al. (2018 [10]; 2019 [11]) demonstrated the dose-dependent cytotoxicity of HAD-B1 in cisplatin-resistant A549 (A549CR) cells and H1975 cells, with IC50 values of 0.41 mg/mL and 0.128 mg/mL, respectively. Notably, cordycepin, a major bioactive component of HAD-B1, showed cytotoxicity with IC50 values of 41.8 μM (A549CR) and 83.6 μM (H1975). In comparison, afatinib (AFT), a second-generation EGFR-TKI, exhibited IC50 values of 2.03 µM (A549CR) and 0.057 µM (H1975). Song et al. (2022) demonstrated that HAD-B1 downregulates MET expression in a dose-dependent manner, potentially mitigating the AR in EGFR-TKIs [12]. Notably, in multiple xenograft mouse models, HAD-B1 plus AFT significantly reduced the tumor burden compared to vehicle control and AFT alone, providing a rationale for the use of HAD-B1 in NSCLC treatment [11,12]

Despite the promising findings of HAD-B1 in preclinical settings, an investigator-initiated trial of HAD-B1 plus AFT did not show improvements in clinical outcomes such as overall survival [13]. This discrepancy underscores the need for a deeper understanding of the exposure–response dynamics of the combination therapy. Model-based approaches provide a quantitative framework to bridge this gap by linking drug exposure with tumor response and elucidate the pharmacological interactions of the combination therapy [14,15]. For instance, Li et al. (2016) developed a PK/PD model of erlotinib and sunitinib in NSCLC xenograft mice, confirming the synergism and identifying optimal dosing via simulation [16]. Similarly, Xiong et al. (2021) applied translational modeling of tepotinib to bridge preclinical and clinical data, supporting phase II dose selection [17].

The present study conducted PK/PD modeling in xenograft mice to determine the therapeutic response relative to drug exposure as well as the PD interactions between AFT and HAD-B1. A mouse PK study was conducted to characterize the PK of AFT in the presence and absence of HAD-B1. A PK model for AFT was subsequently developed and integrated into a tumor growth model, using PD data from a xenograft mouse model treated with either AFT monotherapy or combination with HAD-B1. The PK/PD model characterized the exposure–response relationship and elucidated the synergistic interaction between AFT and HAD-B1. Simulations were conducted to predict tumor response under different treatment groups. The finding of this study provides quantitative insights into the interaction between AFT and HAD-B1, which may support dose optimization strategies in future clinical trials.

## 2. Results

### 2.1. PK Study

#### 2.1.1. PK Analysis

**Figure 1:**
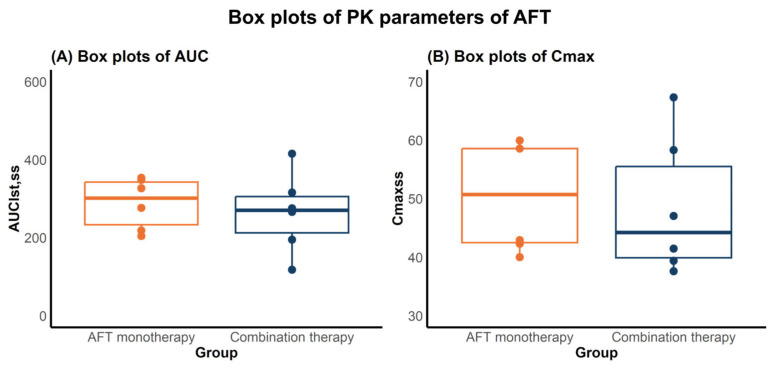
Box plots of PK parameters of AFT in mice following AFT monotherapy or combination therapy with HAD-B1. (**A**) AUC_lst’ss_ and (**B**) Cmax_ss_. Each dot represents a parameter value of an individual animal. Boxes indicate the interquartile range (IQR), with the bold line indicating the median, and whiskers representing 1.5× IQR.

Serum concentrations of AFT in mice were determined following daily doses of AFT 10 mg/kg for 30 days, either alone (AFT monotherapy) or in combination with HAD-B1 400 mg/kg (combination therapy), using a validated Liquid Chromatography–Tandem Mass Spectrometry (LC-MS/MS) method. Noncompartmental analysis (NCA) was performed to calculate the area under the curve from zero to the last measurement at steady-state (AUC_lst,ss_) and the maximum concentration at steady-state (Cmax_ss_) of AFT, both with or without HAD-B1. The AUC_lst,ss_ and Cmax_ss_ values are summarized in Table 1 and illustrated in Figure 1.

No statistically significant differences in AFT PK parameters were observed between the two groups (*n* = 6 per group), as determined by the Mann–Whitney U test (*p* = 0.471). This suggests that coadministration with HAD-B1 may not affect the PK of AFT in mice.

#### 2.1.2. PK Model for AFT

The observed PK data of AFT were analyzed using NONMEM software (7.5.1 version, ICON Development Solutions, Hanover, MD, USA). The PK model for AFT in mice was established as a one-compartment model with first-order absorption and elimination. Table 2 presents the estimated PK parameters of AFT. Diagnostic plots for the PK model are described in Supplementary Materials (Figures S2 and S3).

**Figure 2:**
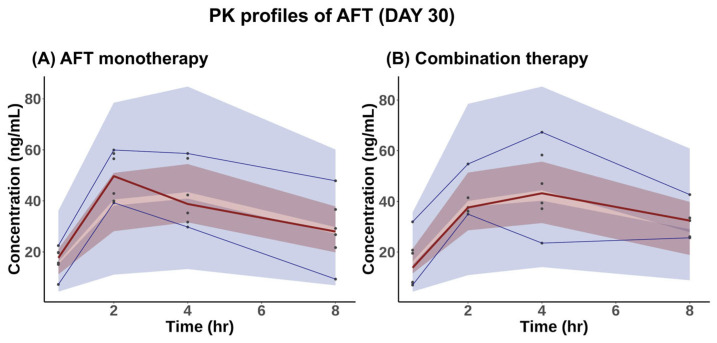
VPCs of serum concentrations of AFT versus time on Day 30 following the administration of AFT with or without HAD-B1 for a consecutive 30 days. (**A**) AFT monotherapy: 10 mg/kg/day AFT and (**B**) Combination therapy: 10 mg/kg/day AFT + 400 mg/kg/day HAD-B1. The red solid line indicates the median of the observed PK data, with the light-red shade indicating the 95% confidence interval of the median predicted concentrations. The blue solid lines indicate the 5th and 95th percentiles of the observed PK data, while the light-blue shade shows the 90% prediction intervals of the predicted concentrations. Black dots are the observed PK data.

Figure 2 illustrates the visual predictive checks (VPCs) of AFT’s serum concentrations over time for both AFT monotherapy and combination therapy. The observed serum concentrations for each group were generally within the prediction intervals at all time points, suggesting that the PK model provides a reliable description of the data.

### 2.2. PK/PD Model

#### 2.2.1. PK/PD Model Development

**Figure 3:**
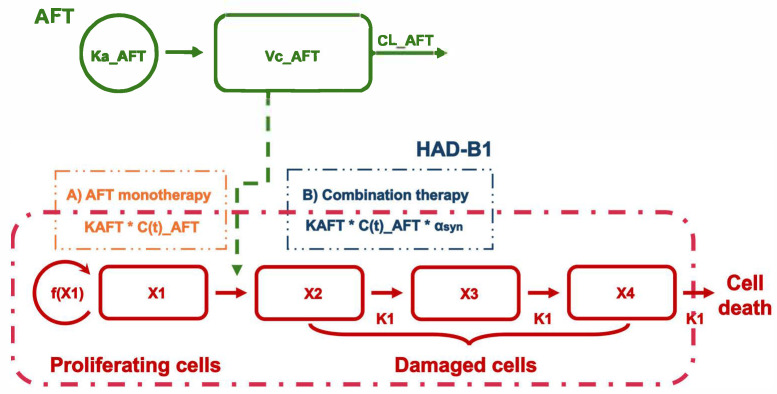
Schematic representation of the final PK/PD model. The PK model for AFT was developed as a one-compartment model with first-order absorption and elimination. CL_AFT and Vc_AFT represent the clearance and volume of distribution of AFT, respectively, and C(t)_AFT denotes the concentrations of AFT over time. The tumor compartment consists of proliferating tumor cells (X1) and damaged cells (X2–X4), which transition through a series of compartments before cell eradication. f(X1) represents the cell proliferation rate, and K_1_ denotes the cell death rate. The drug effect is modeled using K_AFT_, drug potency of AFT, and α_syn_, a synergistic factor representing the effect of HAD-B1 in the combination therapy.

The PK/PD model was developed to characterize the antitumor effects of AFT and its combination with HAD-B1. Figure 3 presents a schematic representation of the developed PK/PD model.

The tumor growth dataset from a xenograft mouse model of gefitinib-resistant HCC827 cells (HCC827-GR) was used to establish the PK/PD model [12]. Table 3 presents the parameter estimates of the PK/PD model. For natural tumor growth, the model structure proposed by Simeoni [18] was utilized to describe the dynamics of proliferating cells based on the tumor volumes in the control group. The exponential growth rate (λ_0_) and linear growth rate (λ_1_) were estimated at 0.156 day^−1^ and 35.5 mm^3^∙day^−1^, respectively. The initial tumor volume was determined to be 21.4 mm^3^.

For treated groups, the λ_0_ and λ_1_ values were fixed based on the control group, and the initial tumor volume was estimated at 32.7 mm^3^. A cell distribution model (CDM), introduced by Simeoni, was applied to characterize the antitumor effects of AFT [19]. In this model, the drug does not exert a direct cytotoxic effect but instead redirects proliferating tumor cells into a death pathway, where they are progressively eliminated through a series of transit compartments. This structure captures the delayed onset of tumor regression relative to drug exposure. A linear model was used to describe the drug effect within the CDM framework, assuming a direct proportionality between AFT concentration and the rate at which tumor cells are committed to death [19]. This process was governed by the potency parameter KAFT, estimated at 0.0034 mL·ng^−1^·day^−1^. The cell death rate constant (K1), reflecting the transit rate through the death compartments, was estimated at 2.2 day^−1^. Additionally, the threshold concentration for tumor eradication (C_T_), derived from the model, was calculated as 46 ng/mL based on the equation C_T_ = λ_0_ / K_AFT_ [18].

Related to combination treatment in the PK/PD model, the influence of HAD-B1 was incorporated into AFT’s potency (K_AFT_), modeled using a synergistic factor (α_syn_) and a binary term (HAD-B1_i_), where HAD-B1_i_ was set to 0 for AFT monotherapy and 1 for the combination therapy group:

K_AFT_ × (1 + α_syn_ × HAD-B1_i_) (1)

α_syn_ was estimated as 0.45, indicating that coadministration of HAD-B1 leads to a 45% increase in tumor inhibition compared to AFT monotherapy.

#### 2.2.2. Model Evaluation

**Figure 4:**
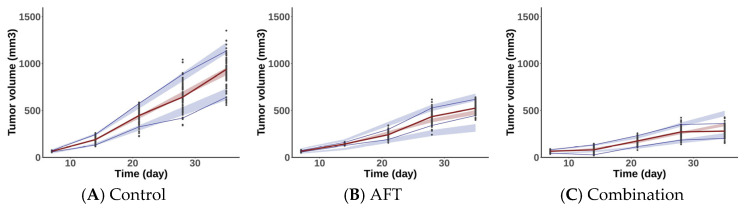
VPC plots of the PK/PD model in xenograft mouse with HCC827-GR. (**A**) Control, (**B**) AFT (10 mg/kg/day), (**C**) Combination: AFT (10 mg/kg/day) plus HAD-B1 (400 mg/kg/day). The red solid line shows the median of the observed tumor volumes, with the light-red shade indicating the 95% confidence interval of the median predicted tumor volumes. The blue solid lines indicate the 5th and 95th percentiles of the observed tumor volumes, while the light-blue shade indicates the 90% prediction intervals of the predicted tumor volumes. Black dots are the observed tumor volume data.

VPCs of the control group and treated groups from the developed PK/PD model are described in Figure 4. As shown in the VPCs, most of the observed datasets fall within the 90% confidence interval, suggesting that the developed model reasonably predict the tumor profiles of the observations. Goodness-of-fit (GOF) plots for the PK/PD model are shown in Figure S4 (Supplementary Materials).

#### 2.2.3. Model-Based Simulations

**Figure 5:**
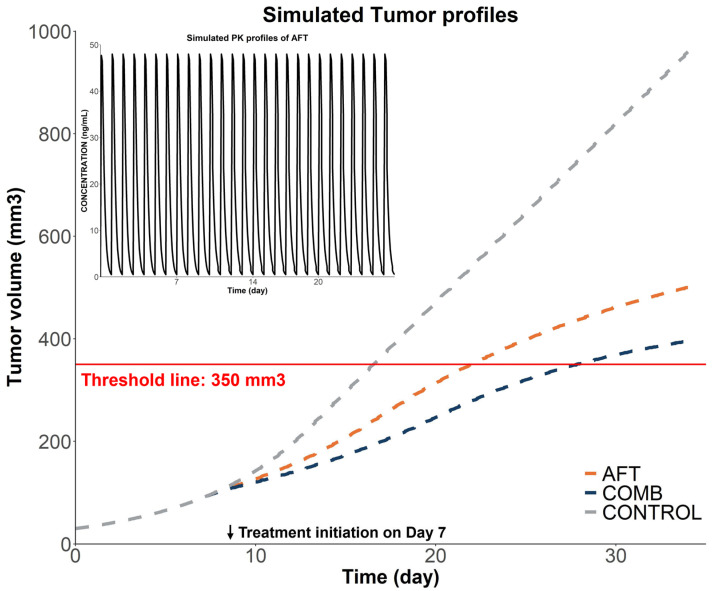
Simulated tumor volumes versus time under different treatment groups. AFT: 10 mg/kg/day of AFT; COMB: 10 mg/kg/day of AFT + 400 mg/kg of HAD-B1 once daily. The solid red line represents a tumor volume threshold of 350 mm^3^ and the dashed line represents the predicted tumor volume profiles for each of the groups. The figure in the upper left corner shows the simulated serum concentration–time profile of AFT at a dose of 10 mg/kg/day. AFT represents AFT monotherapy, COMB represents combination therapy, and CONTROL represents the control group.

Simulations were conducted based on the final PK/PD model to predict and compare tumor responses under the following dosing schedules: the AFT group received 10 mg/kg of AFT once daily, whereas the COMB group received 10 mg/kg of AFT and 400 mg/kg of HAD-B1 once daily. As illustrated in Figure 5, CONTROL, AFT, and COMB groups reached the tumor volume threshold (350 mm^3^) in 16, 22, and 28 days, respectively. The COMB group showed greater tumor inhibition relative to the control and AFT monotherapy groups.

## 3. Discussion

The introduction of EGFR-TKIs has led to significant advancements in NSCLC treatment; however, the inevitable emergence of resistance limits their long-term efficacy. Several combination strategies targeting multiple pathways have been explored to overcome this challenge [20]. HAD-B1, a Korean herbal mixture, has exhibited therapeutic potential as the adjuvant therapy to EGFR-TKIs by enhancing treatment efficacy and attenuating drug resistance in NSCLC. Despite promising preclinical findings, clinical trials have not demonstrated enhanced outcomes of the combination therapy in overall survival or tumor size reduction compared to AFT monotherapy. PK/PD modeling was utilized in this study to bridge the gap between the promising preclinical results and the limited clinical outcomes. We developed the integrated PK/PD model to identify the contribution of AFT to the tumor size reduction and quantitatively explore the therapeutic synergism of the combination treatment. The model-based simulations suggest that the combination was more effective in delaying tumor growth compared to monotherapy, providing insights into its potential benefits and limitations.

Assessing potential PK drug–drug interactions (DDI) is crucial in combination strategies to ensure patient safety and maintain therapeutic efficacy. AFT is an active compound in the combination treatment, known as a narrow therapeutic window in clinical settings [21,22], necessitating a detailed PK characterization in the presence of HAD-B1. As AFT is a substrate of P-glycoprotein (P-gp), the concurrent use of a P-gp inhibitor could increase its exposure [23,24,25]. Among the constituents of HAD-B1, *Panax ginseng* has been reported to modulate the P-gp activity [26]. Given the potential involvement of P-gp mediated pathways, DDI potential warrants further evaluation. In this study, NCA results showed that AUC_lst,ss_ were 287.63 ± 65.36 ng∙h∙mL^−1^ for monotherapy and 263.87 ± 101.78 ng∙h∙mL^−1^ for combination therapy. Similarly, Cmax_ss_ values were 50.37 ± 9.54 ng∙mL^−1^ and 48.50 ± 11.86 ng∙mL^−1^, respectively. Statistical analysis revealed no significant difference in the PK parameters, suggesting that HAD-B1 may not influence the exposure of AFT.

Several tumor growth models, including the Gompertz, Logistic, and Simeoni models, were explored to determine the best fit for the observed tumor growth profiles in the control group. These models describe nonlinear tumor growth, generally transitioning from an initial rapid growth phase to a slower growth phase [18,27]. The natural tumor growth curves of HCC827-GR were best described by the Simeoni model, which accounts for a gradual transition of tumor growth from exponential growth (λ_0_) to linear growth (λ_1_).

The PK/PD model quantitatively characterizes the exposure–response relationship of AFT. The tumor growth rates (λ_0_ and λ_1_) were fixed to the estimates from the control group to serve as the base line for all treated groups [16,18]. The PK/PD model was developed based on the assumption that AFT induces a non-proliferative state in tumor cells, eventually leading to cell death. The linear model was employed to describe the inhibition effect of tumor cells by AFT, which is proportional to both drug concentrations and proliferating tumor size. Considering the delayed cell death post-treatment, the model incorporated three-transit compartments with the cell death rate K_1_. As a secondary parameter, C_T_ was determined to be 46 ng/mL, representing the threshold concentration required for complete tumor regression [18]. According to the model, when steady-state concentration (C_ss_) of AFT remains below C_T_, the tumor reaches a stable size instead of being fully eradicated. Conversely, if C_ss_ exceeds C_T_, complete tumor eradication occurs, regardless of the initial tumor size. In our study, the observed average concentration at steady-state (C_avg,ss_) was 12 ng/mL, calculated using the equation C_avg,ss_ = AUC_ss_/τ, where τ denotes the dosing interval (24 h). This observed concentration is lower than C_T_, suggesting that 10 mg/kg AFT is insufficient to achieve complete tumor eradication. This finding reflects the insensitivity of AFT to HCC827-GR, as demonstrated by in vitro studies (IC50 = 12.87 μM) [12].

HAD-B1 monotherapy showed minimal tumor suppression in the HCC827-GR xenograft model in the prior study [12]. Moreover, the inability to detect its active constituents in mouse serum made it infeasible to construct a standalone PK/PD model for HAD-B1. However, when combined with AFT, HAD-B1 showed greater tumor inhibition compared to the control and AFT monotherapy. For the combination treatment, the contribution of HAD-B1 was incorporated into the PK/PD model as the synergistic factor (α_syn_) modulating AFT’s potency. The developed PK/PD model indicated HAD-B1 augments AFT’s potency by 45%. This synergistic factor quantitatively identifies the combination effect, where a value greater than 1 indicates synergy, a value of 1 represents an additive effect, and a value less than 1 indicates antagonism [28]. In this study, the factor was estimated at 1.45, confirming the therapeutic synergy between HAD-B1 and AFT. The synergy quantified in our PK/PD model may be partly explained by previously reported mechanisms, where HAD-B1 was shown to mitigate resistance to EGFR-TKIs by modulating MET signaling [12]. Although HAD-B1 itself did not affect EGFR phosphorylation, which is significantly inhibited by AFT, it effectively reduced the phosphorylation of MET, reducing the MET expression in a dose-dependent manner in HCC827-GR cells. This complementary mechanism—involving simultaneous inhibition of EGFR (by AFT) and MET (by HAD-B1)—likely underlies the enhanced tumor suppression observed with the combination therapy. Consequently, the combined inhibition of these pathways may disrupt critical downstream signals responsible for cancer cell proliferation and survival, further explaining the synergistic anticancer effect observed in this study.

The model-based simulation aimed to analyze the relative efficacy of each treatment by comparing the time taken for the tumor volume to reach the predefined threshold. The simulation results demonstrated that tumor growth was significantly delayed in the combination treatment group (28 days to reach 350 mm^3^) compared to monotherapy (22 days) or control (16 days). These findings highlight the potential for optimizing dosing regimens to improve clinical outcomes. In the clinical trial [13], the combination therapy group received HAD-B1 at 972 mg/day, equivalent to 200 mg/kg/day in mice by body surface area conversion—half the dose used in our preclinical study. The expected improvements in overall survival and tumor reduction were not observed in this clinical trial, which may be partly attributed to the lower HAD-B1 dose. Therefore, increasing the HAD-B1 dose to levels comparable to the preclinical settings (equivalent to 1944 mg/day in humans) may enhance therapeutic outcomes. Toxicology studies in rats have determined that the no observed effect level (NOAEL) of HAD-B1 exceeds 2000 mg/kg [29], with no mortality, no abnormal clinical signs, no significant changes in body weight, organ weight, or histopathological findings observed at this dose. These results suggest that a higher dose may be feasible in humans. However, this interpretation should be approached with caution, as the dose–response relationship of HAD-B1 has not been fully characterized and safety profiles may vary between species. Further clinical studies are warranted to validate the safety and therapeutic potential of higher HAD-B1 doses.

The present study offers valuable insights. Nevertheless, some limitations of this study should be acknowledged. PK/PD modeling was performed using a single NSCLC cell line. Thus, it may not fully capture the heterogeneity of NSCLC, considering the diverse mechanisms for tumor growth and drug resistance in NSCLC. Multiple NSCLC cell lines must be included in future studies to validate and broaden the applicability of the findings of the present study. The PK/PD model was constrained by the single-dose design and the absence of the standalone HAD-B1 PK/PD component which limited the ability to fully characterize the dose–response relationships of each agent. Furthermore, the PK analysis was based on serum concentrations, and neither AFT nor HAD-B1 levels were directly measured in tumor tissue. As a result, the potential differences in drug behavior at the tumor site were not considered. To address this limitation, future studies should incorporate multiple dosing levels for both agents and include direct measurement of drug concentrations at the tumor site. Such an approach would enable robust estimation of drug-specific effects and support the simulation of optimal dosing strategies. Finally, the model in the present study assumed the synergistic effects without incorporating detailed molecular mechanisms of HAD-B1 related to MET inhibition. A more detailed mechanistic understanding of MET inhibition by HAD-B1 and its integration into the model could enhance its predictive accuracy.

In conclusion, the PK/PD model was successfully developed to quantify the exposure–response relationship and confirm the synergistic interaction of the combination therapy treating NSCLC with EGFR-TKI resistance. To the best of our knowledge, this is the first study to integrate an herbal medicine in combination with an EGFR-TKI into the PK/PD model in xenograft mice. These findings provide insights into PD contribution of HAD-B1 in combination therapy, laying the groundwork for future studies to optimize combination therapies in clinical settings.

## 4. Materials and Methods

### 4.1. Compounds and Reagents

AFT was purchased from the Tokyo Chemical Industry (Tokyo, Japan). Domperidone purchased from Sigma Aldrich (St. Louis, MO, USA) was used as the internal standard in PK analysis. HAD-B1, a blended herbal extract comprising *Panax notoginseng* Radix, *Cordyceps militaris*, *Panax ginseng* C. A. Mey, and *Boswellia carteri* Birdwood, was supplied by the East West Cancer Center (Dunsan Korean Medicine Hospital of Daejeon University, Daejeon, Republic of Korea). The ingredients of HAD-B1 were soaked for 18 h in a bath of distilled water at 60 °C, and the supernatant was obtained. These extracts were concentrated using a rotary vacuum evaporator at 60 °C for 2 h and subsequently dried on a flat evaporator at 60 °C for 8 h and dissolved in distilled water for use in experiments.

### 4.2. PK Study

#### 4.2.1. Animals

Five-week-old female BALB/C nude mice (18–20 g) were purchased from Nara Biotech Co. Ltd. (Pyeongtaek, Republic of Korea). All animal experiments were accordant with the Guide for the Care and Use of Laboratory Animals [30] and were approved by the Institutional Animal Care and Use Committee of Osong Medical Innovation Foundation, Korea (Grant No. KBIO-IACUC-2021-142). Animals were housed in a controlled environment maintained at 22.4 ± 0.3 °C, with 54.1 ± 2.6% humidity, under a 12 h light/dark cycle from 8 AM to 8 PM.

#### 4.2.2. PK Experiment

BALB/C nude mice were randomly divided into two groups (*n* = 6 per group) receiving either AFT alone (10 mg/kg/day) or combination of AFT (10 mg/kg/day) with HAD-B1 (400 mg/kg/day) via oral gavage for 30 days. Blood samples (50 uL) were collected from individual mice at 0.5, 2, 4, and 8 h following AFT administration on the final day (Day 30). The blood samples were collected in serum-separating tubes and centrifuged at 10,000 rpm for 10 min. The serum was stored at −80 °C until analysis. Concentrations of AFT in serum were measured using the validated LC-MS/MS method (details provided in Supplementary Materials).

#### 4.2.3. NCA PK Analysis

NCA was performed using the Noncompart (0.7.0) package in R to calculate PK parameters, such as the AUC_lst,ss_ and the Cmax_ss_. Statistical comparisons of PK parameters between the two groups were performed using the non-parametric Mann–Whitney U test, with significance set at *p* < 0.05.

#### 4.2.4. PK Model for AFT

The PK model for AFT was developed as the one-compartment model with first-order absorption and elimination. The differential equations are as follows:

(2) $${\mathrm{dA}}_{1}(t)/\mathrm{dt}=-\mathrm{Ka}\;\times \;A_{1}\left(t\right)$$

(3) $${\mathrm{dA}}_{2}(t)/\mathrm{dt}=\mathrm{Ka}\;\times \;A_{1}\left(t\right)-\mathrm{Kel}\;\times \;A_{2}\left(t\right)$$

where Ka represents the absorption rate constant, and Kel represents the elimination rate constant, with A_1_(t) and A_2_(t) denoting the deposit and central compartment, respectively. Initial conditions were defined as A_1_(0) = Dose and A_2_(0) = 0.

### 4.3. PK/PD Model

#### 4.3.1. PD Data

The tumor growth datasets from our previous study were used to establish the PK/PD model [12]. HCC827 (1 × 10^6^ cells/head) resistant to gefitinib was inoculated subcutaneously into the right flank of BALB/C nude mice to establish a xenograft mouse model. Tumor dimensions were measured by digital caliper and tumor volumes were calculated by the following equation: Tumor size (mm^3^) = 0.5 × length (mm) × width^2^ (mm^2^). When tumor size reached 80–100 mm^3^, the mice were randomly assigned into four groups (*n* = 6 per group) receiving either vehicle control, AFT alone (10 mg/kg/day), HAD-B1 alone (400 mg/kg/day), or a combination of AFT with HAD-B1 through oral gavage for 30 days. The tumor size and body weight were monitored daily. The inhibitory effects on tumor growth observed in the combination treatment group were significantly greater than those observed in the control and monotherapy groups (*p* < 0.05). The data are presented as mean and standard deviation (SD). For PK/PD modeling, tumor growth profiles (*n* = 100) were generated based on the mean and SD values, assuming that tumor size follows a normal distribution.

#### 4.3.2. PK/PD Model and Simulations

The integrated PK/PD model was sequentially established. The natural tumor growth model was developed to characterize the tumor growth dynamics of HCC827-GR in the control group. The PK profiles of AFT were simulated based on the PK model and integrated into the tumor growth model to quantify the exposure–response relationship and synergistic interaction in combination therapy. The tumor datasets from different treatment groups were pooled together to develop the final PK/PD model

Regarding natural tumor growth, various models were tested to fit the tumor growth curves of HCC827-GR, including the Gompertz, Logistic growth, and Simeoni model. The Simeoni model was selected as it exhibited the best adherence to the tumor profiles observed in the vehicle group, characterized by initial exponential growth followed by a linear phase [18]. This model assumed there is a threshold tumor size (Wth), at which tumor growth switched from exponential to linear phase. The differential equations are as follows:

(4) $$dX_{1}\left(t\right)/\mathrm{dt}=\lambda_{0}\;\times {\;X}_{1}\left(t\right),\;X_{1}\left(t\right)\;\leq \;W_{\mathrm{th}}$$

(5) $$dX_{1}\left(t\right)/\mathrm{dt}=\lambda_{1},\;X_{1}\left(t\right)\;>\;W_{\mathrm{th}}$$

(6) $$X_{1}(0)=V_{0}$$

where V_0_ represents the tumor size at the inoculation time (t = 0). W_th_ could be expressed as a function of λ_0_ and λ_1_ ensuring the continuity of the derivatives of the model (Equations (4) and (5)) at X_1_(t) = Wth:

(7) $$\lambda_{0}\;\times \;W_{\mathrm{th}}=\lambda_{1}$$

For computational reasons, Equations (4)–(6) could be conveniently expressed in a single differentiable function as follows:

(8) $$dX_{1}\left(t\right)/\mathrm{dt}=\lambda_{0}\;\times \;X_{1}(t)\;/\;{\left[1+{(\frac{\lambda_{0}}{\lambda_{1}}\;\times \;X_{1}\left(t)\right)}^{\varphi }\right]}^{1/\varphi }$$

(9) $$X_{1}(0)=V_{0}$$

where φ is a shape factor, fixed at 20 according to the prior study [18].

For treated groups, the concentrations of AFT were simulated based on the PK model and integrated into the tumor growth inhibition model. λ0 and λ1, estimated from the natural tumor growth model, were fixed for the treated groups. The linear model was utilized to describe the effects of AFT, proportional to both drug concentrations and tumor size. The model assumed the damaged tumor cells by AFT eradicated progressively using the three-transit compartments model with cell death rate K_1_. The contribution of HAD-B1 was incorporated as $\alpha_{\mathrm{syn}}$ in the model to describe the synergistic effect in the combination treatment. The differential equations for the final PK/PD model are as follows:

(10) $$dX_{1}\left(t\right)/\mathrm{dt}=\lambda_{0}\;\times \;X_{1}(t)\;/\;{\left[1+{(\frac{\lambda_{0}}{\lambda_{1}}\;\times \;X_{1}\left(t)\right)}^{\varphi }\right]}^{1/\varphi }-\;K_{\mathrm{AFT}\;}\times \;(1+\alpha_{\mathrm{syn}}\;\times \;\mathrm{HAD}\text{-}B1_{i})\;\times \;C(t)\;\times \;X_{1}\left(t\right)$$

(11) $$dX_{2}\left(t\right)/\mathrm{dt}=K_{\mathrm{AFT}\;}\times \;(1+\alpha_{\mathrm{syn}}\;\times \;\mathrm{HAD}\text{-}B1_{i})\;\times \;C(t)\;\times \;X_{1}\left(t\right)-K_{1}\times \;X_{2}\left(t\right)$$

(12) $$dX_{3}\left(t\right)/\mathrm{dt}=K_{1}\times \;X_{2}\left(t\right)-K_{1}\times \;X_{3}\left(t\right)$$

(13) $$dX_{4}\left(t\right)/\mathrm{dt}=K_{1}\times \;X_{3}\left(t\right)-K_{1}\times \;X_{4}\left(t\right)$$

(14) $$V(t)=X_{1}\left(t\right)+X_{\;2\;}\left(t\right)+X_{3}\left(t\right)+X_{4}\left(t\right)$$

(15) $$X_{1}\left(0\right)=V_{0}$$

(16) $$X_{2}(0)=X_{3}(0)=X_{4}(0)=\;0$$

where X_1_(t) denotes proliferating cells. C(t) represents concentrations of AFT over time. X_2_(t), X_3_(t), and X_4_(t) represent transit compartments of the damaged cells being processed through eradication phases, and V(t) represents the total volume of tumor cells. Initial conditions were defined at the time of tumor cell inoculation (t = 0), with V_0_ representing the baseline tumor volume. Treatment was initiated at t = 7 days, once tumors reached approximately 80–100 mm^3^.

Model-based simulations using the final PK/PD parameters were conducted to predict tumor growth trajectories and assess the relative efficacy of each treatment regimen by comparing the time required for the tumor volume to reach the predefined threshold.

#### 4.3.3. Model Development and Evaluation

Model development and simulations were performed using a nonlinear mixed-effects modeling approach through NONMEM software. The first-order conditional estimation method was applied throughout the model-building process. The residual variability was modeled using a proportional error structure. Model selection was based on achieving the minimum objective function value and the precision of parameter estimates, assessed by relative standard errors. To evaluate the model, GOF plots were utilized, and VPCs were performed with 1000 simulation replicates, generating prediction intervals for 5th, 50th, and 95th percentiles, which were then used to compare against the observed data.

## 5. Conclusions

In conclusion, this study developed a PK/PD model to quantitatively characterize the interaction between HAD-B1 and AFT in the xenograft mouse model of NSCLC. To the best of our knowledge, this is the first study to integrate herbal medicine in combination with an EGFR-TKI into a PK/PD framework to describe tumor growth inhibition. The model successfully captured the exposure–response relationship and confirmed a synergistic antitumor effect of the combination therapy. These findings provide quantitative support for the pharmacological rationale behind combining HAD-B1 with EGFR-TKIs and may guide dose optimization in future clinical studies.

## Figures and Tables

**Table 1:** PK parameters (Mean ± SD) of AFT in mice derived using NCA.

Parameters	AFT Monotherapy	Combination Therapy
AUC_lst,ss_ (ng∙mL^−1^∙h)	287.63 ± 65.36	263.87 ± 101.78
Cmax_ss_ (ng∙mL^−1^)	50.37 ± 9.54	48.50 ± 11.86

**Table 2:** Parameter estimates of the PK model for AFT.

Parameters	Unit	Estimates	RSE (%)	Shrinkage (%)
Ka	h^−1^	0.2	39	
CL/F	L∙h^−1^∙kg^−1^	24.5	9	
Vd/F	L∙kg^−1^	56.1	17	
**Inter-individual variability (IIV)**				
IIV on CL/F	%	30.9	20	17
IIV on Vd/F	%	50.2	19	14
**Residual variability**				
Prop.ER	%	29.0	9	

Ka, first-order absorption rate constant. CL/F, the apparent clearance of AFT. Vd/F, the apparent volume of distribution. IIV, inter-individual variability. Prop.ER, proportional error. RSE, relative standard error.

**Table 3:** Parameter estimates of the PK/PD model.

Parameters	Control Group	Treated Groups
λ_0_ (day^−1^)	0.156	0.156 (FIX)
λ_1_ (mm^3^∙day^−1^)	34.9	34.9 (FIX)
φ	20 (FIX)	20 (FIX)
V_0_ (mm^3^)	21.4	32.7
K_1_ (day^−1^)	–	2.2
K_AFT_ (mL∙ng^−1^∙day^−1^)	–	0.0034
α_syn_	–	0.45
**Residual variability**		
Prop.ER	0.169	0.215

λ_0_, exponential growth rate. λ_1_, linear growth rate. φ, shape factor. K_1_, cell death rate. K_AFT_, drug potency. α_syn_, synergistic factor.

## Data Availability

The datasets used in this study are available from the corresponding author upon reasonable request.

## Supplementary Materials

The following supporting information can be downloaded at: https://www.mdpi.com/article/10.3390/ph18050748/s1, Figure S1: Calibration curve of AFT in mouse serum, Figure S2: Combined GOF plots of the PK model for AFT, Figure S3: VPCs of the PK model for AFT, Figure S4: Combined GOF plots of the PK/PD model, Figure S5: HPLC profile of major components in HAD-B1; Table S1: QC sample analysis of AFT in mouse serum
