# Supplementary material for: Model-Based Evaluation of HangAmDan-B1 and Afatinib Combination Therapy in HCC827 Xenograft Mice with Resistance to Epidermal Growth Factor Receptor Tyrosine Kinase Inhibitor
Source: Pharmaceuticals (Basel). 2025 May 19;18(5):748. doi: 10.3390/ph18050748 (PMC12114871; doi:10.3390/ph18050748)
Supplement: Supplementary file 1 [file pharmaceuticals-18-00748-s001.zip › pharmaceuticals-3591387-supplementary.pdf]

Model-Based Evaluation of HangAmDan-B1 and Afatinib Combination Therapy in HCC827 Xenograft Mice with Resistance to Epidermal Growth Factor Receptor Tyrosine Kinase Inhibitor

Supplementary Materials

Sung-yoon Yang [1,†], Lien Thi Ngo [2,3,†], Soyoung Lee [1,†], Hwi-yeol Yun [1,4,5,†], Tham Thi Bui [1,6], Dong-Hyeon Kim [7], Jung-woo CHAE [1,4,5,*] and Sojung Park [7,8,*]

1 College of Pharmacy, Chungnam National University, Daejeon 34134, Republic of Korea; 201851000@o.cnu.ac.kr (S.-y.Y.); sy.lee@cnu.ac.kr (S.L.); hyyun@cnu.ac.kr (H.-y.Y.); bttham@hpmu.edu.vn (T.T.B.)
2 Faculty of Pharmacy, PHENIKAA University, Yen Nghia, Ha Dong, Hanoi 12116, Vietnam; lien.ngothi@phenikaa-uni.edu.vn
3 PHENIKAA Research and Technology Institute (PRATI), A&A Green Phoenix Group JSC, No. 167 Hoang Ngan, Trung Hoa, Cau Giay, Hanoi 11313, Vietnam
4 Department of Bio-AI convergence, Chungnam National University, Daejeon 34134, Republic of Korea
5 Senior Health Convergence Research Center, Chungnam National University, Daejeon 34134, Republic of Korea
6 Faculty of Pharmacy, Haiphong University of Medicine and Pharmacy, Haiphong 180000, Vietnam
7 Department of Internal Medicine, Pusan National University, Korean Medicine Hospital, Yangsan 50612, Republic of Korea; dongxian92@gmail.com
8 Department of Korean Internal Medicine, School of Korean Medicine, Pusan National University, Yangsan 50612, Republic of Korea
* Correspondence: jwchae@cnu.ac.kr (J.-w.C.); vivies@hanmail.net (S.P.)
† These authors contributed equally to this work as co-first authors.

Table of contents

1. LC-MS/MS method to determine the serum concentrations of AFT in mice 3

2. PK Model Development and Evaluation 6

3. PK/PD mode Evaluation 9

4. HPLC profiles for components of the Herbal Mixture 10

1. LC-MS/MS method to determine the serum concentrations of AFT in mice

Serum concentrations of AFT in mice were measured using the high-performance liquid chromatography (1200 series HPLC; Agilent, Santa Clara, CA, USA) coupled to mass spectrometry (Qtrap 4000; Sciex, Framingham, MA, USA). AFT was separated by XTERRA reverse-phase C18 column (50 x 2.1 mm, 3 μm; Waters, Milford, MA, USA), maintained at 20 °C. The mobile phase was composed of water with 0.1% formic acid (A) and methanol with 0.1% formic acid (B) at a flow of 0.4 mL/min in gradient elution. The mass spectrometry analysis was conducted with an electrospray ionization probe in the positive ion mode. The ion spray voltage was 5500 V, and the source temperature was 500 °C. Multiple reaction monitoring transitions of each analyte were m/z 486.1 → 371.1 for AFT and m/z 426.3 → 175.1 for domperidone (DOMP). The retention times for AFT and DOMP were 3.7 and 3.65, respectively. Calibration standard samples were prepared to set up calibration range, and Quality control (QC) samples were run in triplicate to test accuracy of the developed method. Calibration curve for AFT was linear in the range of 5–500 ng/mL ($R^2 > 0.99$). The lower limit of quantification was determined to be 5 ng/mL. The mean concentrations of QC samples were within 15% of the nominal values.

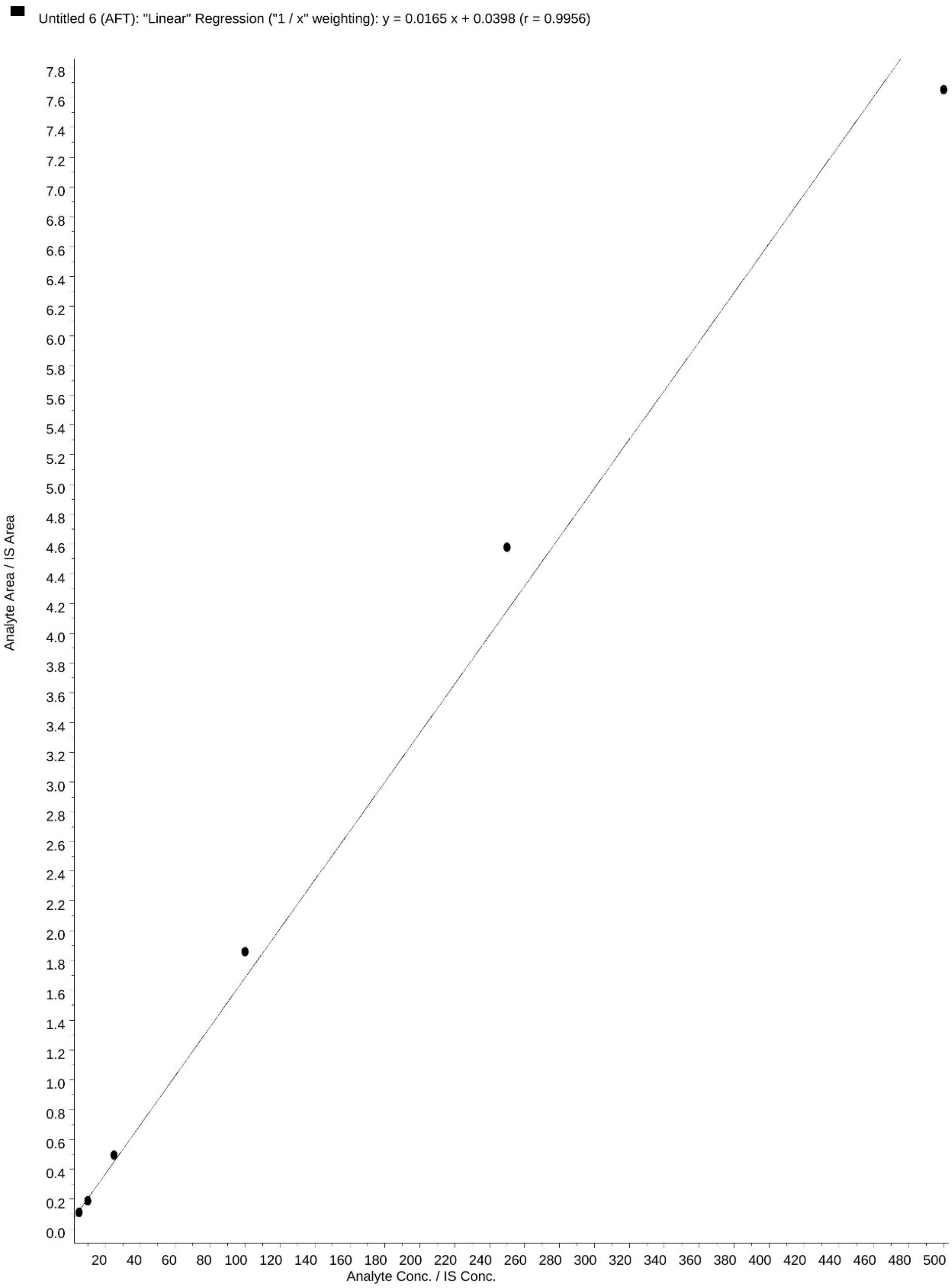

Figure S1. Calibration curve of AFT in mouse serum. The calibration curve was constructed using a weighted (1/x) linear regression and showed excellent linearity over the range of 5–500 ng/mL ($R^2 > 0.99$).

Table S1. QC sample analysis of AFT in mouse serum

Sample Name	Analyte Peak Area	IS Peak Area	Area Ratio	Calculated Conc (ng/mL)	CV (%)
QC1_1 (15 ng/mL)	6.71E+04	2.30E+05	2.92E-01	15.27	1.79
QC1_2 (15 ng/mL)	6.42E+04	2.33E+05	2.76E-01	14.29	4.75
QC1_3 (15 ng/mL)	6.71E+04	2.65E+05	2.53E-01	12.93	13.77
QC2_1 (200 ng/mL)	8.06E+05	2.28E+05	3.54E+00	211.84	5.92
QC2_2 (200 ng/mL)	8.22E+05	2.28E+05	3.61E+00	216.09	8.04
QC2_3 (200 ng/mL)	8.24E+05	2.61E+05	3.16E+00	188.93	5.54
QC3_1 (400 ng/mL)	1.65E+06	2.38E+05	6.93E+00	417.76	4.44
QC3_2 (400 ng/mL)	1.61E+06	2.38E+05	6.76E+00	407.57	1.89
QC3_3 (400 ng/mL)	1.65E+06	2.83E+05	5.83E+00	350.94	12.26

2. PK Model Development and Evaluation

The structure model for AFT's PK was assumed to follow the first-order absorption and elimination. The first-order conditional estimation with the interaction method was applied to each step of the model development for AFT. Inter-individual variability was tested for each parameter, and residual variability was modeled using a proportional error structure. The final model was selected based on the decrease in objective function value, the precision of parameter estimates, and goodness-of-fit (GOF) diagnostics. The selected model was then evaluated by visual predictive checks (VPCs).

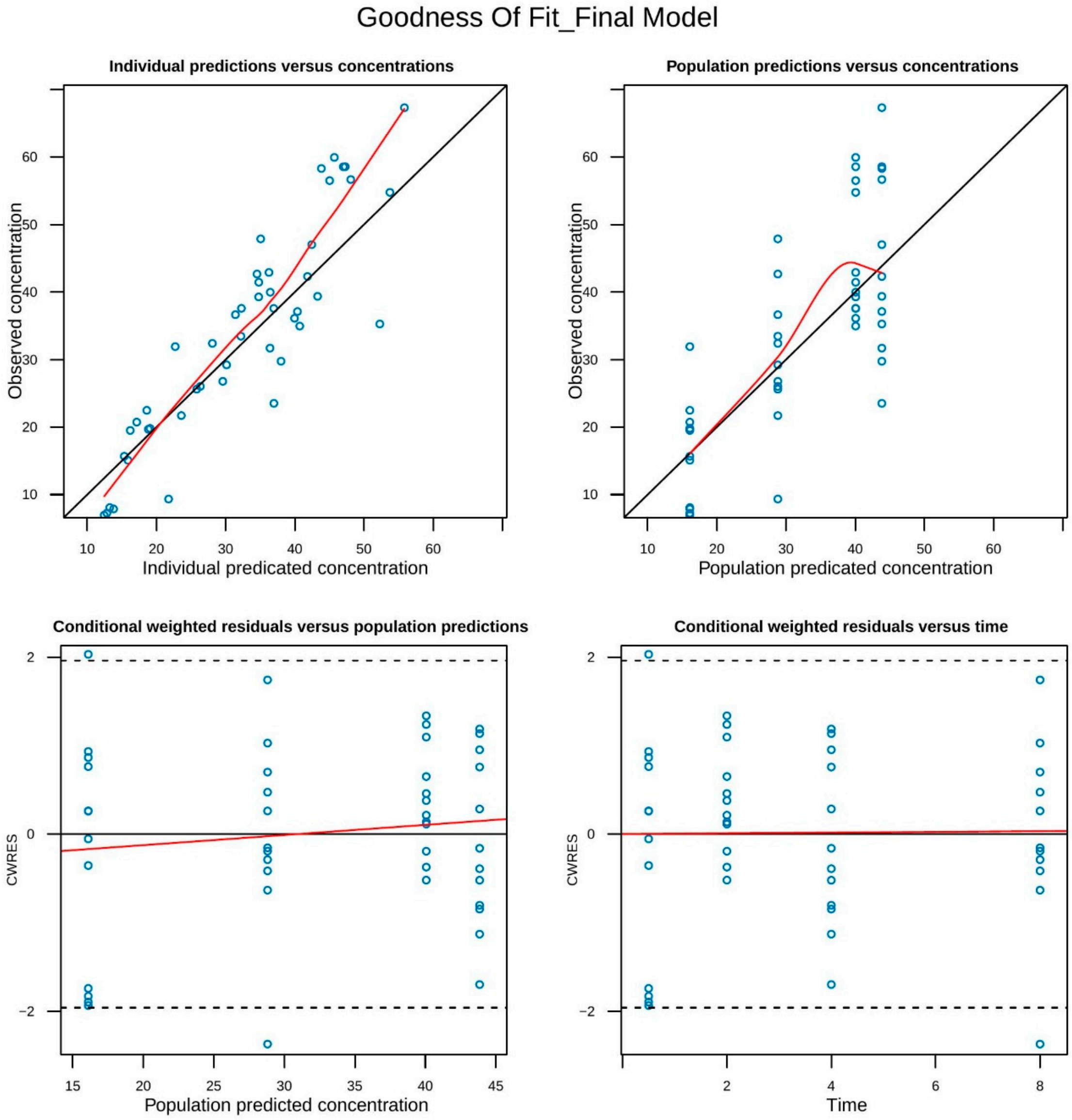

Figure S2. Combined GOF plots of the PK model for AFT. CWRES, conditional weighted residual.

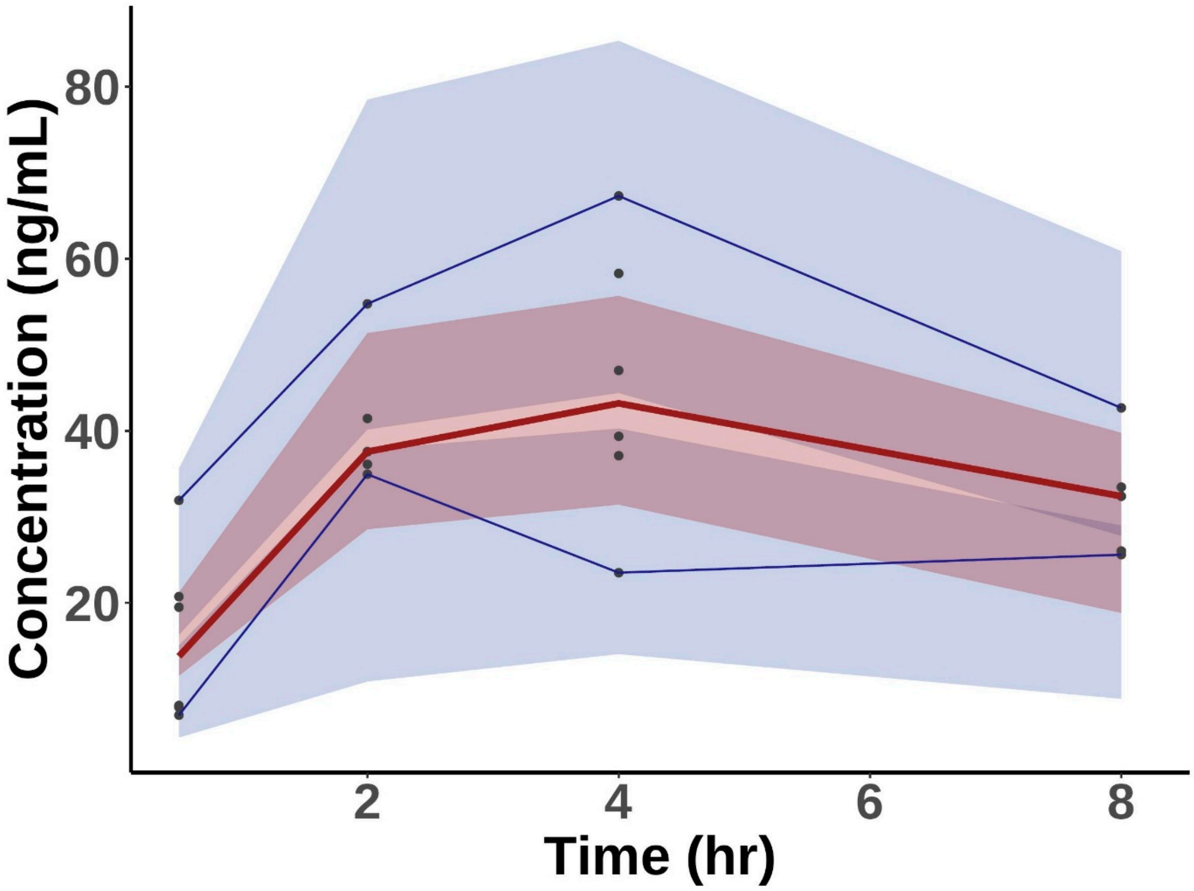

Figure S3. VPCs of the PK model for AFT. Black dots represent the observed concentrations; red solid line is the median of the observed concentrations; light blue shade is 90% prediction intervals of the predicated concentrations, and red shade is 95% confidence interval of the median predicated concentrations.

3. PK/PD mode Evaluation

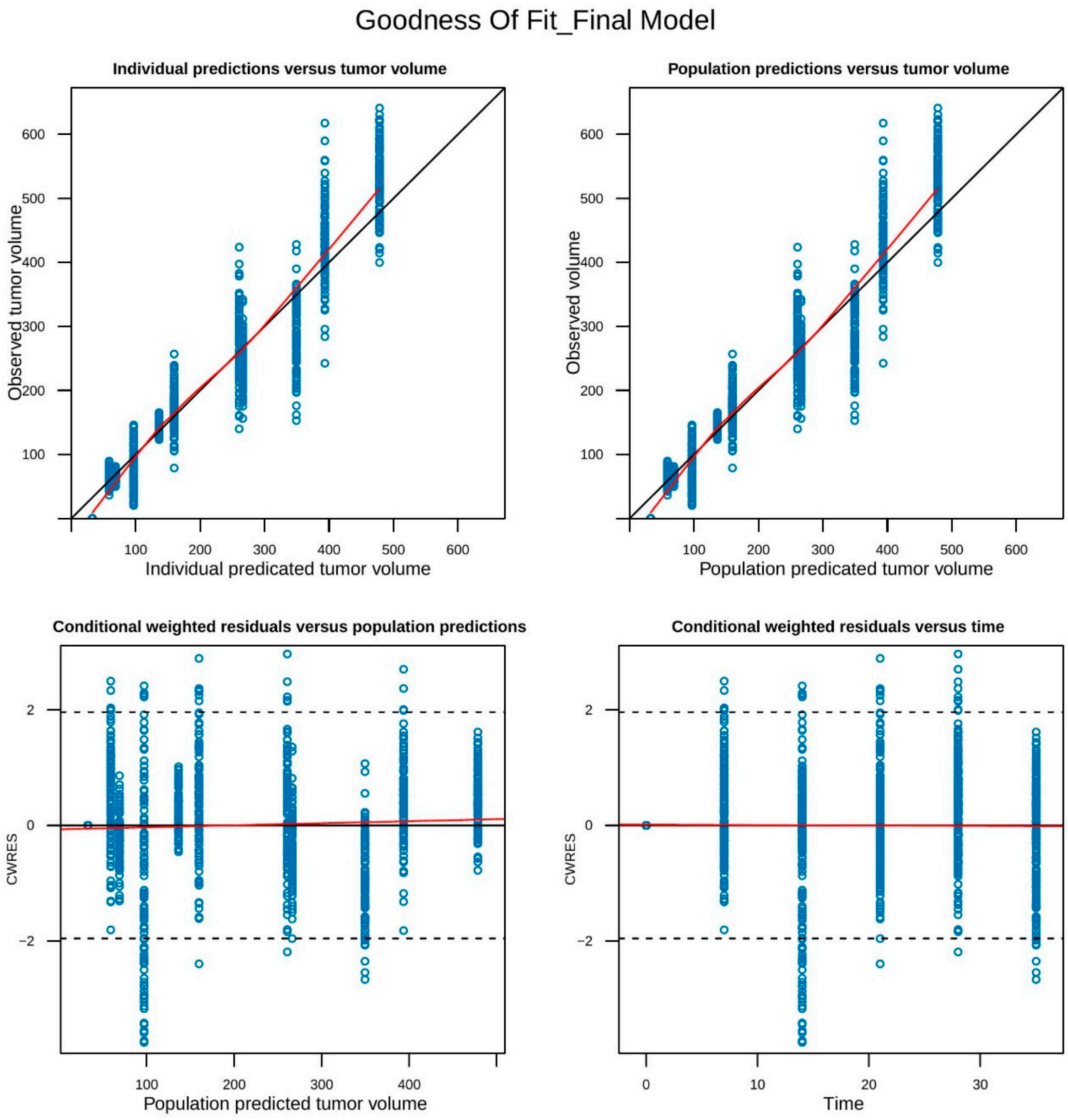

Figure S4. Combined GOF plots of the PK/PD model. CWRES, conditional weighted residual.

4. HPLC profiles for components of the Herbal Mixture

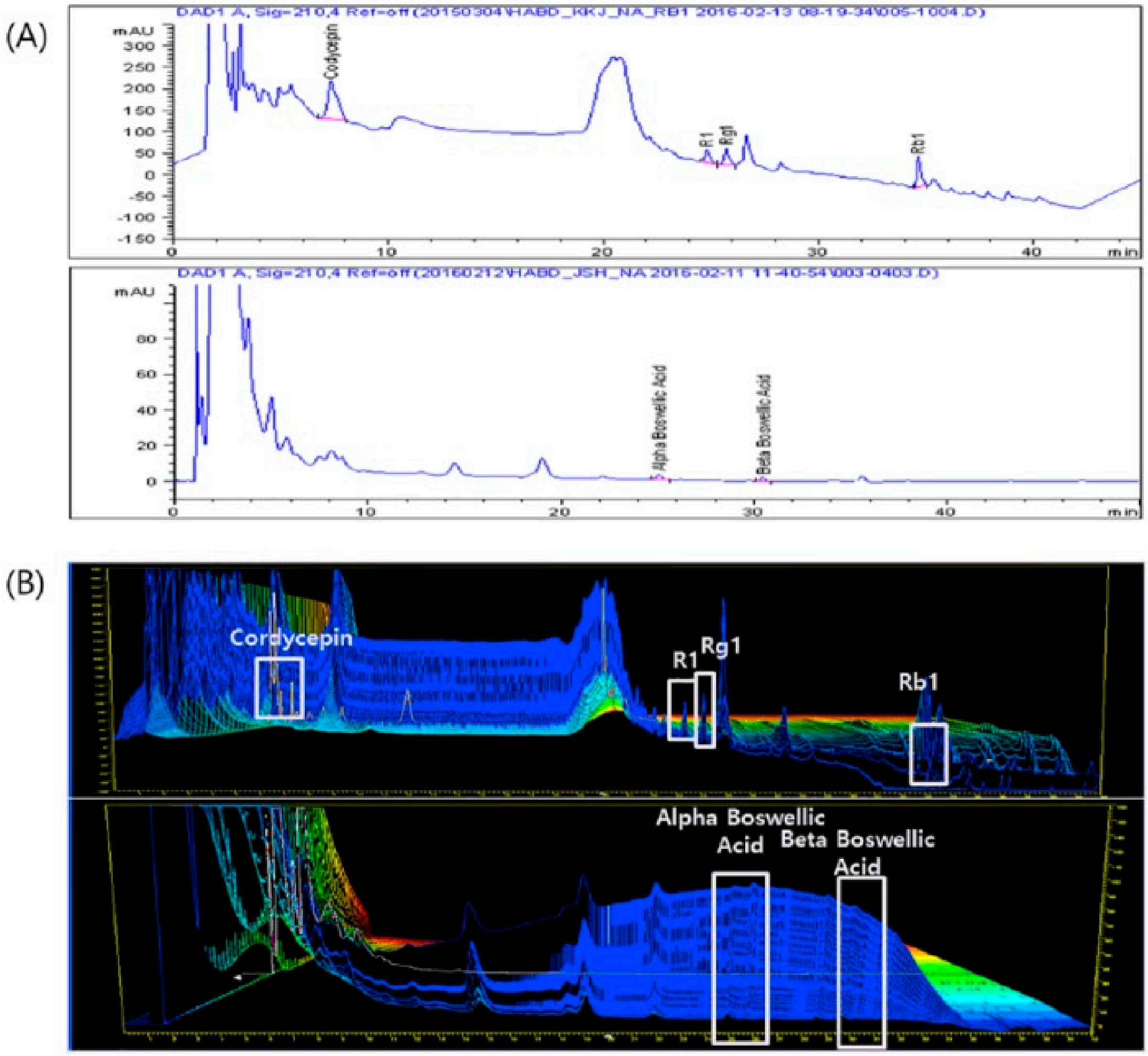

Figure S5. HPLC profile of major components in HAD-B1. For the quantitative analysis of 1 tablet of HAD-B1, methanol extract of HAD-B1 was applied to the octadecylsilylated silica gel column on HPLC and eluted by acetonitrile mixed with distilled water (A). The 3-dimensional HPLC profile of HAD-B1 (B). HAD-B1 detected the presence of 6 compounds: cordycepin, R1, Rg1, Rb1, α-boswellic acid, and β-boswellic acid.
